# Assessment of some Herbal Drugs for Prophylaxis of Peptic Ulcer 

**Published:** 2014

**Authors:** Ahmed A Gohar, Ahmed A Zaki

**Affiliations:** a *Pharmacognosy Department, Faculty of Pharmacy, Mansoura University, Mansoura 35516, Egypt. *

**Keywords:** Anti-ulcer agents, Indomethacin-induced ulcer, Gastro-protection, Herbal remedies

## Abstract

Aqueous (hydrophilic) and chloroform (Lipophilic) extracts of nine medicinal plants currently used in Egyptian traditional medicine to treat some gastrointestinal tract (GIT) disorders were tested for their gastro-protective effect against the incidence of peptic ulcer. Indomethacin-induced ulcer in a rat model was used for this testing. *Mentha microphylla*, *Brassica oleracea *Capitata (Cabbage), *B. oleracea *Botrytis (cauliflower) aqueous fraction, Portolaca oleracea polysaccharide fraction, *Oreganum marjoranum*, *Matricaria recutita*, *Solanum nigrum *hydrophilic and lipophilic fractions, in addition to the chloroform fraction of *Portolaca oleracea *and *Cicorium intybus *afforded high protection against the incidence of gastric ulcer (~95%). *O. syriacum *hydrophilic and lipophilic fractions and gum arabic afforded moderate prophylactic effect. *L. sicerarea*, *C. intybus *hydrophilic fractions and *M. microphylla *lipophilic fraction were inactive. Herbs represent excellent resources for cost-effective and readily available gastro-protective remedies without side effects.

## Introduction

Peptic ulcer is a popular disease all over the world. It is the most common gastrointestinal tract (GIT) disorder in clinical practice. Recent survey revealed that 1.84% of population in the United States, 2.7% in Australia, and about 1.8% in Canada, Great Britain and Egypt suffer from this disease. The mortality rate, which has decreased modestly in the last few decades, is approximately 1 death per 100,000 cases (1-2). The etiology of the peptic ulcer and principles of treatments have been discussed by different authors (3-5). 

The drugs currently used in the treatment of gastric ulcers are antacids, anticholinergics, proton pump inhibitors and H_2_-receptor antagonists (6-7). However, the majority of these drugs produce adverse reactions, such as: hypersensitivity, arrhythmia, impotence, gynecomastia and hematopoietic changes (8- 9). Despite progress in conventional chemistry and pharmacology in producing effective anti-ulcer drugs, the plant kingdom might provide a useful source of new compounds that are used for development as pharmaceutical entities or, alternatively, as simple dietary adjuncts to existing therapies. Several herbs and spices have been used to treat GIT disorders, including gastric ulcers (3-5, 10-13). The early search in the area of medicinal plant in the treatment of peptic ulcers opened the discovery of the first drug effective against peptic ulcer; carbenoxolone from *Glycyrrhiza glabra *(14-16), and licorice root fluid extract were used to treat stomach ulcers in patients had not improved with conventional medication. The glycyrrhizin of licorice was found to stop two enzymes that break down prostaglandin E (17). Effectiveness of other plant resources as cabbage in improving peptic ulcers have been reported (18-20).

The aim of the present study is the assessment of antiulcerogenic and/or gastro-protective effects of some of herbal extracts currently used in Egyptian folk medicine for some of GIT disorders.

## Experimental

*Animals*

Female Wister rats (175-200 g), were maintained on standard pellet diet and water ad libitum under standard conditions of 12 h dark-12 h light, humidity (60 ± 1.0%) and temperature (21 ± 1 °C). They were acclimatized to laboratory condition for seven days before commencement of the experiments. Fasting for 24 h was used prior to all assays because tested drugs were always administered orally by gavage (5). The experimental protocols were approved by the Institutional Animal Care and Use Committee; faculty of pharmacy, Mansoura University, Egypt.

*Plant materials*

*Mentha microphylla, Oreganum marjoranum, O. syriacum, Solanum nigrum, Chicorium intybus, Matricaria recutita *(Geraman chamomile)*, Brassica oleracea *(white cabbage and cauliflower)*, Lagenarea sicerarea, *and *Portolaca oleracea *were collected from wild and cultivated plants in Dakahlia area. Identity of the collected plants was confirmed by Professor I. Mashaly, department of systematic botany, Faculty of Science, Mansoura University. Voucher specimens were deposited in pharmacognosy department, Faculty of Pharmacy, Mansoura University, Egypt.

*Extract preparation*

The polysaccharide fraction of fresh *P. oleracea *was prepared by blending 500 g of fresh herb in an electric mixer with distilled water (1 L) then lift overnight. The viscid suspension was filtered through glass wool followed by precipitation with ethanol. The precipitated polysaccharide fraction was washed with acetone, dried under vacuum and reserved frozen for testing.

500 g fresh *B. oleracea *Capitata (white cabbage) and the same weight of *B. oleracea *Botrytis (cauliflower) were separately homogenized with 1 L distilled water and left overnight. Solvents were, separately, evaporated under vacuum in rotary evaporator. The prepared extracts were reserved frozen for testing. Gum Arabic was used as pharmaceutical grade (El-Nasr Pharmaceutical Co., Egypt). 

Another 500 g fresh *B. oleracea *Capitata (white cabbage) and the same weight of *B. oleracea *Botrytis (cauliflower) were extracted with boiling methanol (1 L, each) in a soxhlet apparatus. Air dried and powdered 200 g of *M. microphylla, O. marjoranum, O. syriacum, S. nigrum, C. intybus, M. recutita*, and *L. cicerarea *were separately extracted with hot methanol (3 x 500 mL) in a soxhlet apparatus. The solvents were, separately, evaporated under vacuum in rotary evaporator and the residue, of each plant sample, was suspended in 200 mL distilled water and partitioned with chloroform (3 x 100 mL). The combined chloroform extracts and the mother aqueous liquors were then evaporated under vacuum in rotary evaporator and residues were stored frozen for biological testing. 

The crude extracts were suspended in normal saline, with the aid of tween 20 at the dose levels of 200 mg/Kg just before testing. Mucilage of *P. oleracea *polysccaride and gum Arabic were macerated in normal saline at the dose levels of 20 mg/Kg, 1 h before testing.

*Ulcer induction with indomethacin and testing the samples*

Animals, for each test, were divided into four groups of six rats each. All the animals were fasted for 24 h before the experiment. First group served as negative control and received distilled water, while second group served as positive control and received Cimetidine (250 mg/Kg). Third and fourth groups served as test groups and administered the tested sample by gastric gavage 200 mg/Kg, respectively. After 1 h of drug treatment, Indomethacin (30 mg/Kg, p.o.) was given (21-22). After 2 h, rats were anesthetized with ether and sacrified by cervical dislocation then the abdomen was opened, stomach excised and opened along the greater curvature, stomach contents were collected in graduated tubes to measure its volume, then stomach was washed with normal saline, and fixed in formalin. Lesions in the glandular part of the stomach were measured under an illuminated magnifying microscope (10X). Long lesions were counted and measured along their greater length in mm unit. Ptecheal lesions were counted and each five lesions were taken as 1 mm of ulcer (23). The ulcerative lesion (dark red lines) index of each animal was scored and the percentage protection was calculated according to Scarlat *et al*., 1985 (24). The experiment was repeated, following the same sequence, for each tested plant material.

*Statistical analysis*

The results were expressed as mean ± SEM. The individual data of each test and the control groups were submitted to one-way ANOVA with the level of significance set at *P *˂ 0.05 (Instat-2 Computer Program: GraphPad Software Inc., V2.04, San Diego, CA, USA)

## Results and Discussion

The p.o. administration of indomethacin at dose of 30 mg/Kg was sufficient to induce gastric ulcers in rats. The score of indomethacin-induced ulcer was 50.17 ± 0.47 mm and the secretions in the stomach were about 2.5 ± 0.2 mL (negative control). Oral administration of various tested plant materials before indomethacin treatment lowered the ulcer score, with some fractions, up to100% reduction (Table 1). Some plant extracts showed moderate reduction, while others were not active to protect against the gastric ulceration. Cimetidine, the known H_2_-histamine receptor antagonist was used as a positive control and showed good protection (100% reduction) against indomethacin-induced gastric ulceration (Table 1).

The results of the different experiments run for the assessment of some herbal drugs currently used for some GIT disorders are shown in Table 1. Most of the tested fractions showed a marvelous protection (~95%) as the aqueous and chloroform fractions of *O. marjoram*, *M. recutita, S. nigrum*, *M. microphylla*, *B. oleracea *Capitata (white cabbage), *B. oleracea *Botrytis (cauliflower) aqueous fractions, *P. oleracea *polysaccharide in addition to *C. intybus*, *L. siceraria *chloroform fractions and the total aqueous extract of *B. oleracea *(white cabbage). On the other hand, the aqueous extract of *C. intybus*, *L. siceraria *and the chloroform fraction of *M. microphylla *were not active as ulcer prophylactic extracts. *O. syriacum *extracts showed moderate effect however the aqueous one was more effective than the chloroform fraction. Gum Arabic also showed moderate protection. No gastric secretions were measured for some tested fractions (Table 1) because it was very few suggesting inhibited gastric secretions. The stomachs of rats treated with chamomile aqueous fraction showed full distension with gases.

**Table 1:** Ulcer score, Protection ratio and Volume of secretions in the stomachs of rats

**Screened-plant**		**Ulcer score ** **(mm)**	**Protection ratio (%)**	**Secretion volume (mL)**
*Mentha microphylla *:	Aqueous Chloroform	00.0 ± 0.0^a^ 53.83 ± 2.22^b^	100.00 00.00	--- 4 ± 0.2
*Cichorium intybus*:	Aqueous Chloroform	60.40 ± 1.44^a^ 1.97 ± 1.0^a^	00.00 96.07	5 ± 0.2 ---
*Lagenaria siceraria*:	Aqueous Chloroform	46.97 ± 1.52^c^ 2.67 ± 0.17^a^	6.38 94.67	3.4 ± 0.36 ---
*Oreganum syriacum*:	Aqueous Chloroform	9.00 ± 0.33^a^ 17.3 ± 0.32^a^	82.06 65.51	3.0 ± 0.5 ---
*Oreganum marjoram*:	Aqueous Chloroform	1.01 ± 0.05^a^ 00.00^a^	97.98 100.00	--- ---
Matricaria chamomilla:	Aqueous* Chloroform	00.00^a^ 00.00^a^	100.00 100.00	--- ---
*Solanum nigrum*:	Aqueous Chloroform	00.00 a 00.00 a	100.00 100.00	3.2 ± 0.15 ---
*Brassica oleracea *Capitata, Cabbage:	Aqueous Chloroform Total aqueous	2.26 ± 0.08 a 3.20 ± 0.07 a 1.74 ± 0.07 a	95.49 93.62 96.53	--- ---
*Brassica oleracea *Botrytis, Cauliflower:	Aqueous Chloroform Total aqueous	1.36 ± 0.06 a 00.00 a 8.5 ± 0.21 a	97.28 100 83.05	--- --- ---
*Portolacca oleracaea *polysaccharide		00.00 a	100.00	---
Gum arabic		12.42 ± 0.23^a^	75.24	---
Indomethacin		50.17 ± 0.47	00.00	2.5 ± 0.2
Cimetidine		0.08 ± 0.05^a^	99.84	---

* The stomachs were full of gases;

a : *P *˂ 0.001,

b : *P *˂ 0.05,

c : *P *> 0.05

Chamomile was previously reported as protective for peptic ulcer disease (25). The aqueous extract of chamomile decreases the gastric secretions and acidity so, increases the curative ratio of gastric ulcer (26). *O. marjoram *is a common hot drink and *P. oleracea *is an edible plant whose anti-ulcerative qualities add to their house use as functional food with high medicinal values. The aqueous extract of *P. oleracea *was previously reported as antiulcerogenic principle (27-29). The aqueous fraction of *M. microphylla *showed a prominent protective effect (100% protection), which suggests the beneficial use of infusion or decoction teas of *Mentha *as protective against peptic ulcers. The *Mentha *leaves were previously reported to improve pain of nonulcer dyspepsia (30). However, *Mentha *extract significantly decreases the total acidity in the stomach, it doesn’t affect the volume of gastric juice (31). The total alcoholic extract of *O. syriacum *was previously reported to have 78% protection against ethanol-induced ulcer (8). Both *B. oleracea *white cabbage and cauliflower fractions showed good protection against peptic ulcers, 93.6 – 96.5% and 83.30 -97.3%, respectively. This confirmed the previously reported anti-ulcer effects of these plants due to increased hexosamine levels and antisecretory effect, suggesting gastric mucosal protection (18, 32). While the chloroform extract of *C. intybus *showed 96% protection, it᾽s water soluble fraction increased the ulcer score however it has anti-*Helicobacter Pylori *effect (33). The titled plant roots and leaves were previously reported to have antiulcerogenic effect (34). The aerial parts of *S. nigrum*, powder-form and methanolic extract, were reported to decrease the ulcer index significantly due to inhibition of acid and pepsin secretions (32). The aqueous and ethanolic extracts of *P. oleracea *were previously reported to exhibit gastroprotective effect due to decreased gastric secretions (35).

The development of peptic ulcers can be due to imbalance between the insulting effect of acid and pepsin and the protective effect of mucosal barrier (36). Cimetidine has little cytoprotective effect but its principal action is selective antagonism of H_2_ histaminic receptors and it possesses antisecretory action (37-39). It was reported to inhibit the indomethacin-induced ulcer by 94.8% (40). Other mechanisms are now hypothesized for this imbalance including the decreased gastric hexosamine level and weakness of gastric barrier (10) generation of oxygen free radical and increased peroxidation of the biological membranes (41). Indomethacin is a non-selective cyclooxygenases inhibitor known to induce gastric damage through suppression of prostaglandin generation, overproduction of leukotrienes, topical irritancy and reducing the local blood flow (42). In the stomach, prostaglandins play a vital protective role, stimulating the secretion of bicarbonate and mucus, maintaining mucosal blood flow, and regulating mucosal cell turnover and repair in addition to its action as cytoprotective (43, 44). Drugs which produce prophylaxis against the indomethacin effect can act through antagonizing all or some of its mechanisms. 

## References

[1] Cai S, García Rodríguez LA, Massó-González EL, Hernández-Díaz S (2009). Uncomplicated peptic ulcer in the UK: trends from 1997 to 2005. Aliment Pharmacol. Ther.

[2] Leontiadis GI, Sreedharan A, Dorward S, Barton P, Delaney B, Howden CW, Orhewere M, Gisbert J, Sharma VK, Rostom A, Moayyedi P, Forman D (2007). Systematic reviews of the clinical effectiveness and cost-effectiveness of proton pump inhibitors in acute upper gastrointestinal bleeding. Health Technol. Assess.

[3] Borrelli F, Izzo A (2000). Plant kingdom as a source of anti-ulcer remedies. Phytother. Res.

[4] Al Mofleh IA (2010). Spices, herbal xenobiotics and the stomach: Friends or foes? World J. Gastroenterol.

[5] Sakat SS, Tupe P, Juvekar A (2012). Gastroprotective effect of Oxalis corniculata (whole plant) on experimentally induced gastric ulceration in Wistar rats. Indian J. Pharm. Sci.

[6] Bighetti AE, Antonio MA, Kohn LK, Rehder VL, Foglio M, Possenti A, Vilela L, Carvalho JE (2005). Anti-ulcerogenic activity of a crude hydroalcoholic extract and coumarin isolated from Mikania laevigata Schultz Bip. Phytomed.

[7] Lakshimi V, Singh N, Shrivastva S, Mishra SK, Dharmani P, Palit G (2009). Gedunian and photogedunin of Xilocarpus granatum show significant antisecretory effects and protect the gastric mucosa of peptic ulcer in rats. Phytomed.

[8] Alkofahi A, Atta AH (1999). Pharmacological screening of the anti-ulcerogenic effects of some Jordanian medicinal plants in rats. J. Ethnophrmacol.

[9] Chan FK, Leung WK (2002). Peptic ulcer disease. Lancet.

[10] Akhtar AH, Ahmed KU (1995). Anti-ulcerogenic evaluation of the methanolic extracts of some indigenous medicinal plants of Pakistan in aspirin-ulcerated rats. J. Ethnopharmacol.

[11] Singh S, Majumdar DK (1999). Evaluation of the gastric antiulcer activity of fixed oil of Ocimum sanctum (Holy Basil). J. Ethnopharmacol.

[12] Perera LS, Ruedas D, Gomez BC (2001). Gastric antiulcer effect of Rhizophora mangle L. J. Ethnopharmacol.

[13] Luiz-Ferreira A, Cola M, Barbastefano V, Hiruma-Lima CA, Santos LC, Vilegas W, Brito AR (2012). Antiulcerogenic activity of the aqueous fraction of Anacardium humile St Hil (Anacardiaceae).. J. Med. Plants Res.

[14] Henmann FD (1970). Inhibition of peptic activity by carbenoxolone and glycerrhetinic acid. Gut.

[15] Baron JH (1977). Effect of carbenoxolone sodium on human gastric acid secretion. Gut.

[16] Ali AM, Al-Alousi L, Sae-lem HA (2005). Licorice: A possible anti-inflammatory and Anti-ulcer drug. AAPS Pharm. Sci. Tech.

[17] Shibata S (2000). A drug over the millennia: pharmacognosy, chemistry, and pharmacology of licorice [review]. Yakugaku Zasshi.

[18] Adami E, Marzzi-Uberti E, Turba C (1964). Pharmacological research on gefarnate, a new synthetic isoprenoid with antiulcer action. Arch. Int. Pharmacol. Therap.

[19] Best R, Lewis DA, Nasser N (1984). The antiulcerogenic activity of unripe plantain banana (Musa spp). Brit. J. Pharmacol.

[20] Goel RK, Gupta S, Shankar R, Sanyal AK (1986). Anti-ulcerative effect of Banana powder (Musa sapientum var paradisiacal) and its effect on mucosal resistance.. J. Ethnopharmacol.

[21] Morimoto Y, Shimohara K, Oshima S, Takayoki S (1991). Effects of new anti-ulcer agent KB-5492 on experimental gastric mucosal lesions and gastric mucosal defensive factors, as compared to those of teprenone and cimetidine. Jp. J. Pharmacol.

[22] Muriel PB, Marivane L, Edson LM, Mateus FL, João PB, Jairo KB, Sérgio FA (2008). Evaluation of antiulcer activity of the main phenolic acids found in Brazilian Green Propolis. J. Ethnopharmacol.

[23] Cho CH, Ogle CW (1979). Cholinergic-mediated gastric mast cell degranulation with subsequent histamine H1- and H2-receptors activation in stress ulceration in rats. Europ. J. Pharmacol.

[24] Scarlat M, Sandor VI, Tomas M, Cuparencu P (1985). Experimental anti-ulcet activity of Veronica officinalis L. extract. J. Ethnopharmacol.

[25] McKay DL, Blumberg, JB (2006). A review of the bioactivity and potential health benefits of chamomile tea (Matricaria recutita L). Phytother. Res.

[26] Rezq AA, Elmallh MM (2010). Anti-ulcer effect of cinnamon and chamomile aqueous extractsin rat models. J. Am. Sci.

[27] Ghazanfar SA, Al-Sabahi AM (1993). Medicinal plants of northern central Oman (Arabia). Econom. Bot.

[28] Ezekwe MO, Omara AR, Membrahtu T (1999). Nutritive characterization of purslane accessions as influenced by planting date. Plant Foods for Human Nutrition (Dordrecht).

[29] Bucciarelli AY, Skilair MI (2007). Plantas medicinales de Argentina con actividad Gastroprotectora. Ars. Pharm.

[30] Thompson CJ, Ernest E (2002). Systemic review: herbal medicinal plants for non-ulcer dyspepsia. Aliment. Pharmacol. Ther.

[31] Atta AH, Nasr SM, Monier SM (2005). Antiulcerogenic effect of some plants extracts. Nat. Prod. Radiance.

[32] Akhtar MS, Munir M (1989). Evaluation of the gastric antiulcerogenic effects of Solanum nigrum, Brassica oleracea and Ocimum basilicum in rats. J. Ethnopharmacol.

[33] Uz-Zaman R, Akhtar MS, Khan MS (2006). In-vitro antibacterial screening of Anethum graveolens L fruits, Chicorium intybus L. leaves, Plantago ovate L. seed husk and Polygonum viviparum L. root extract against Helicobacter pylori.. Int. J. Pharmacol.

[34] Jamal A, Siddiqui A, Tajuddin, Jafri MAA (2006). Review on gastric ulcer remedies used in Unani system of medicine. Nat. Prod. Radiance.

[35] Karimi G, Hosseinzadeh H, Ettehad N (2004). Evaluation of the gastric antiulcerogenic effects of Portulaca oleracea L. extracts in mice. Phytother. Res.

[36] Venables CW (1986). Mucus, Peppsin and Peptic Ulcer. Gut.

[37] Hung CR, Lee CH (1991). Protective effect of cimetidine on tannic acid-induced gastric damage in rats. J. Pharm. Pharmacol.

[38] Lozeva V, Marazova K, Belchea A (1994). Histamine and the gastrointestinal tractGastric histamine content and ulcer formation in rats with ethanol-induced injury. Effect of cinnarizine and flunarizine.. Agents action.

[39] Bishayee A, Chattarjee M (1993). Protective effect of Mikania cordata root extract against physical and chemical factors-induced gastric erosions in experimental animals. Planta Med.

[40] Queiroga CL, Silva GF, Dias PC, Possenti A, de Carvalho JE (2000). Evaluation of the antiulcerogenic activity of friedelan-3B-ol and friedlin isolated from Maytenus ilicifolia (Celasteraceae). J. Ethnopharmacol.

[41] Cholbi MR, Paya M, Alcaraz MJ (1991). Inhibitory effects of phenolic compounds on CCl4-induced microsomal lipid peroxidation. Experientia.

[42] Dajani EZ, Agrawal NM (1995). Prevention and treatment of ulcer induced by nonsteroidal anti-inflammatory drugs: an update. J. Physiol. Pharmacol.

[43] Robert A (1979). Cytoprotection by prostaglandin. Gastroenterol.

[44] Hayllar J, Bjarnason I (1995). NSAIDs, COX-2 inhibitors, and the gut. Lancet.

